# Supplementary material for: Feasibility of fresh frozen human cadavers as a research and training model for endovascular image guided interventions
Source: PLoS One. 2020 Nov 30;15(11):e0242596. doi: 10.1371/journal.pone.0242596 (PMC7704126; doi:10.1371/journal.pone.0242596)
Supplement: S1 Table — Gender and age of the donated specimens. (DOCX) [file pone.0242596.s001.docx]

**S1 Table: Baseline characteristics of body donors.** Gender and age of the donated specimens.

|  | Torsos  (n = 6) | Lower extremities  (n=23) | Total  (n=29) |
| --- | --- | --- | --- |
| Sex (male) | 4 (66.7%) | 18 (78.2%) | 22 (75.9%) |
| Age (mean ± SD) | 77.7 ± 5.4 | 73.6 ± 8.7 | 74.4 ± 8.3 |
| SD = standard deviation | | | |
