# Supplementary material for: Feasibility of fresh frozen human cadavers as a research and training model for endovascular image guided interventions
Source: PLoS One. 2020 Nov 30;15(11):e0242596. doi: 10.1371/journal.pone.0242596 (PMC7704126; doi:10.1371/journal.pone.0242596)
Supplement: S2 Table — Table summarizing target vessel cannulation success in all specimens, including information on angiographic visibility of the target vessel and calcification of the target vessel. (DOCX) [file pone.0242596.s002.docx]

**S2 Table: Target vessel cannulation success.**

**S2A Table: Target vessel cannulation success in the fresh frozen human torsos.** Table summarizing target vessel cannulation success of the torsos, including the visibility of the target vessel on angiography and visible calcifications.

| Target Vessel | Cannulation success* | Patent on angiogram † | Visible calcification‡ |
| --- | --- | --- | --- |
| Celiac Artery  (n = 6) | 100% | 100% | 0% |
| Superior Mesenteric Artery  (n = 6) | 100% | 100% | 0% |
| Right Renal Artery  (n = 6) | 100% | 100% | 33.3% |
| Left Renal Artery  (n = 6) | 100% | 100% | 16.7% |
| *defined as: successful cannulation of the artery of interest with guidewire.  †defined as: contrast-enhanced arterial lumen of the artery of interest. At least 5cm of the artery of interest should be visible.  ‡defined as: moderate to severely calcified regions near the ostium of the target vessel visible on preprocedural computed tomography imaging. | | | |

**S2B Table: Target vessel cannulation success in the fresh frozen human lower extremities.** Table summarizing target vessel cannulation success of the lower extremities, including the patency of the artery on angiography and the presence of visible calcifications.

| Target Vessel | Cannulation success* | Patent on angiogram † | Visible calcification‡ |
| --- | --- | --- | --- |
| Posterior Tibial Artery  (n = 23) | 91.3% | 91.3% | 66.7% |
| Anterior Tibial Artery  (n = 23) | 95.6% | 91.3% | 65.2% |
| Peroneal Artery  (n = 23) | 87.0% | 82.6% | 69.6% |
| *defined as: successful cannulation of the artery of interest with guidewire.  †defined as: contrast-enhanced arterial lumen of the artery of interest. At least 5cm of the artery of interest should be visible.  ‡defined as: moderate to severely calcified regions near the ostium of the target vessel visible on preprocedural computed tomography imaging. | | | |

**S2C: Example imaging.** To visually demonstrate the definitions of ‘patent on angiogram’ and ‘visible calcification’ provided in S2A and S2B Tables.

**
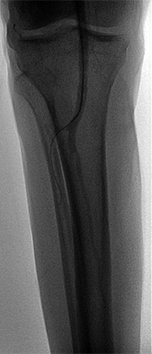
**

**S2C.1 Fig:** **Target vessel cannulation success.** Example of successful cannulation of the anterior tibial artery in a lower extremity.

**
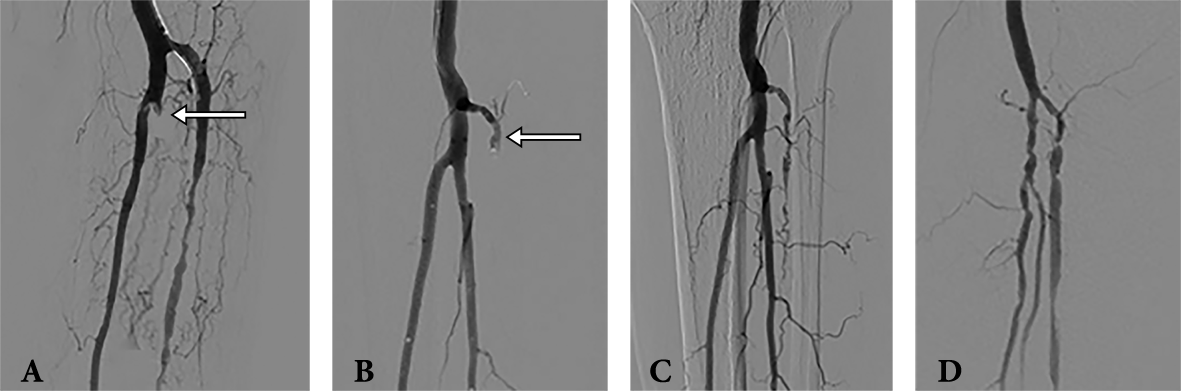
**

**S2C.2 Fig: Patency of target vessel on angiography. Panel A:** An example of a lower extremity in which the peroneal artery (white arrow) would be rated as ‘occluded on angiography’, while the anterior and posterior tibial arteries would be rated as ‘patent on angiography’. **Panel B:** An example of a lower extremity in which the anterior tibial artery (white arrow) would be rated as ‘occluded on angiography, while the posterior tibial artery and peroneal artery would be rated as ‘patent on angiography’. **Panel C** **and D:** Examples of lower extremities in which all three target vessels would be rated as ‘patent on angiography’.

**
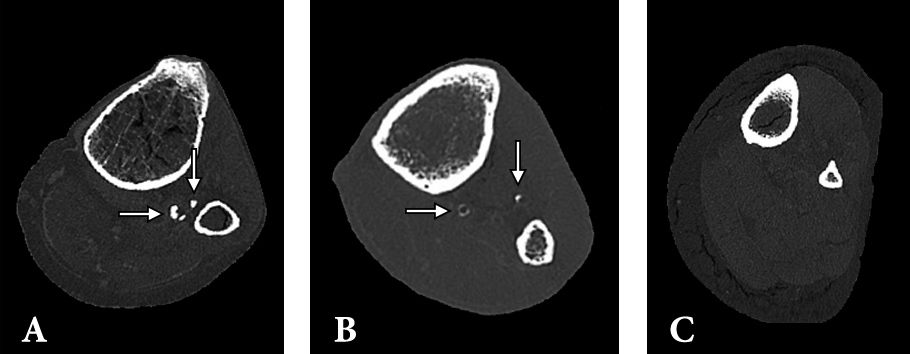
**

**S2C.3 Fig: Arterial calcifications in the lower extremities visible on preprocedural computed tomography.** These computed tomography scans are acquired several weeks to months before the procedure, with the lower extremities in frozen condition. **Panel A:** An example of a lower extremity with severe calcification of the anterior tibial artery and the tibioperoneal trunk. This example has been rated as ‘visible calcification’ of all three target vessels. **Panel B:** An example of a lower extremity with moderate calcification in the anterior tibial artery and the posterior tibial artery. This example has been rated as ‘visible calcification’ of the anterior and posterior tibial artery, while the peroneal artery is rated as ‘no visible calcification’. **Panel C:** An example of a lower extremity without visible calcification. This example is rated as ‘no visible calcification’ of all three target vessels.
