# Supplementary material for: Feasibility of fresh frozen human cadavers as a research and training model for endovascular image guided interventions
Source: PLoS One. 2020 Nov 30;15(11):e0242596. doi: 10.1371/journal.pone.0242596 (PMC7704126; doi:10.1371/journal.pone.0242596)
Supplement: Attachment — Submitted filename: PONE-D-20-02240 - Response to Reviewers Rev2.docx [file pone.0242596.s004.docx]

Dear editor and reviewers,

Thank you for your comments based upon the first revision of the manuscript PONE-D-20-02240. In this second revision we have thoroughly revised the supporting information, as elaborated on in the comments below.

We look forward to hearing from you regarding this submission. We would be glad to respond to any further questions and comments that you may have.

On behalf of all authors,

Marloes Jansen, MSc.

PhD-candidate Vascular Surgery

**Review Comments to the Author**:

**Reviewer #2:**

Reviewer #2: This reviewer does not have further comments. The manuscript was corrected based on our suggestions and questions.

**Response**: Thank you for your suggestions.

**Reviewer #3:**

Reviewer #3: The manuscript was well revised and I have only minor comments as follows.

1. Regarding the supporting information, the excel files should be summarized using tables, not raw data (e.g., how many cases were documented as ‘Angiographically visible’ etc…). Also, please provide the abbreviations list for the supporting information.

2. I think if supporting information would be refined, it would be nice information for the readers. Please define the definitions for ‘Angiographically visible’ and ‘calcification visible’.

**Response**: Thank you for your suggestions. Based on your comments we have revised the supporting information files. Firstly, we deposited the underlying (raw) data at a public data depository (Mendeley Data) as suggested in Plos One’s publication guidelines. The data is accessible with the DOI provided in the reference section. [line 262-263 of the redline document].

Secondly, both supporting information files (S1 and S2) now contain summarization tables, rather than raw data. Both files contain more elaborate background information including a table legend, spelled out abbreviations and definitions.

We reframed the term ‘angiographically visible’ to ‘patent on angiogram’. And provided definitions of the terms ‘patent on angiogram’ and ‘visible calcifications’ in the tables.
Moreover, we have included several example images, to provide a more visual understanding of these definitions. These images include illustration(s) of 1) ‘target vessel cannulation success’, 2) ‘patent on angiogram’ and 3) ‘calcification visible’. [S1 Tables and S2 Tables]

To convey this information to the readers, we referred to the S2 table in the manuscript. [line 183 – 188 of the redline document]

“*Cannulation of the posterior tibial artery, anterior tibial artery and peroneal artery was successful in 20 (91%), 21 (95%) and 19 (86%) specimens respectively. All six cases of cannulation failure were attributed to high grade stenosis or occlusions of the target vessel, as confirmed by their lack of patency on DSA imaging or, when CT-imaging was available, by presence of local arterial calcifications. More information on these methods can be found in Supporting Information S2.*”
